# Assessment of nutritional status among older people seeking health care in tertiary care hospitals in Mangalore

**DOI:** 10.1186/s12877-026-07950-5

**Published:** 2026-07-03

**Authors:** Pracheth Raghuveer, Mithun Rao, Dayasagar Reddy, Khushi Sunil, Roshna Narayan, Shreya Bera, Prajwal Kolkar, Rakshita Moolya

**Affiliations:** 1 Department of Epidemiology, National Institute of Mental Health and Neuro Sciences, Bengaluru, India; 2 Department of Community Medicine, Kasturba Medical College Mangalore, Manipal Academy of Higher Education, Manipal, India; 3 Kasturba Medical College Mangalore, Manipal Academy of Higher Education, Manipal, India

**Keywords:** Older, Geriatrics, Malnutrition, Sustainable Development Goals

## Abstract

**Background:**

As the proportion of the geriatric population has increased worldwide, increases in mortality and morbidity are inevitable. To ensure healthy aging, preventive measures must be taken at the earliest stage. SDG 2.2 aims to overcome malnutrition, including malnutrition, among the older population. Thus, this study was designed to assess the prevalence of malnutrition among older people who visit hospitals for various ailments so that suggestive measures can be advised.

**Methods:**

The study was conducted after approval from the Institutional Ethics Committee (IEC) and permissions from the administration of the institution. Older people aged above 60 years who provided consent to participate were considered for the study, while those who were not in a position to provide responses were excluded from the study. A sample of 117 participants was interviewed using a semistructured validated questionnaire consisting of sociodemographic details and the MNA-SF questionnaire. The collected data were entered and then analysed using IBM SPSS (ver 29).

**Results:**

A total of 69 (59%) patients received health care from government hospitals, whereas 48 (41%) patients sought health care from private hospitals, with almost equal participation from both genders. One-third of them had hypertension and diabetes mellitus. Upon screening, 40.2% of the older people had a normal nutritional status, whereas the rest of them were either at risk or already having malnutrition. With 58.1% of the participants being malnourished or at risk of malnourishment in the 60–69 age group, among the participants in the lower/upper lower socioeconomic status category, 62.9% were malnourished or at risk of malnutrition, while 56.4% were in the lower middle/upper middle socioeconomic status category.

**Conclusion:**

The present study highlights the substantial burden of malnutrition among older individuals. Two-thirds of the individuals were either at risk of developing malnutrition or had already developed malnutrition. As none of the sociodemographic factors were found to be associated, qualitative studies are needed to explore the various other reasons for developing malnutrition.

**Supplementary Information:**

The online version contains supplementary material available at 10.1186/s12877-026-07950-5.

## Introduction

As the global population is growing daily, the older population is experiencing proportional growth, which is a hallmark of the 21st century and directly and indirectly affects developed and developing nations worldwide. The population of the older people who are above 60 years of age is projected to cross 1.5 billion by 2050, as per the reports, with the major increase occurring in developing and developed countries, which may face difficulty in handling the growing geriatric population [1]. This shift in the demographic cycle is attributed mainly to the ever-improving health care, resulting in the prevention of morbidity and mortality caused by serious conditions, improvement in sociocultural factors and a trend toward declining fertility rates, which in turn leads to major humongous gains in life expectancy among the population [2]. Across the globe, there has been a large mix of various social policies, financial conditions and healthcare systems owing to adaptations to changes in age and sex structure. Specifically, developing and developed countries present intricate challenges in providing better health infrastructure and social security to the geriatric population, which requires innovative solutions to address modern problems [1–3]. 

As a person grows older, a set of physical, psychosocial and physiological changes occur, which makes him or her vulnerable to health-related conditions, thus making him or her prone to communicable and noncommunicable diseases or lifestyle diseases [3]. Changes in the efficiency of body organs also make them vulnerable to disease conditions. The gradual loss of digestive system efficiency may decrease the hunger, thirst and appetite of a person, which, along with other imbalances during the geriatric age, can lead to nutritional deficiencies [3–6]. Along with psychosocial stressors and mental health, physical inability further deteriorates the range of movements, and decreases in memory, vision and other dental issues become substantial hurdles in trying to access and consume a needed age-appropriate balanced diet, further leading to malnutrition [5, 7, 8]. 

Thus, an imbalance of diet in terms of both quality and quantity along with the health challenges faced by older people may lead to malnutrition worldwide, especially in developing countries in India. In addition to the diet per se, an interplay of various other sociocultural factors, such as food insecurity, poverty, financial constraints and natural decline of abilities, the person may still become vulnerable. A decline in nutritional status may further lead to deterioration of the ecosystem, thus increasing the susceptibility to infections, prolonged wound healing time, and worse outcomes after any disease, thus leading to a vicious cycle of malnutrition and disease progression [3, 9, 10]. 

Although the burden of the disease condition is known among the geriatric population, the attention received among the policy think tanks has been negligible. As a result, older individuals are often left out when nutritional programs are formulated or discussed in a forum, which highlights persistent research gaps in developing and developed countries. This barrier includes not only awareness among health care staff but also low resources to overcome geriatric problems. Thus, to overcome these challenges, ground studies are needed to fill these knowledge gaps and provide evidence for future interventions and policies that can break the vicious cycle of the malnutrition–disease cycle and meaningfully improve the health and quality of life for the growing older population worldwide. Thus, this study was designed to assess the prevalence of malnutrition among older people seeking health care in tertiary care hospitals attached to a medical college in Mangalore and to understand the factors associated with the risk of malnutrition among them.

## Materials and methods

The study was initiated after formal approval from the Institutional Ethics Committee (IEC) and permissions from the medical college and the studied hospitals were obtained. This study was conducted in the outpatient and inpatient departments of two tertiary care hospitals (one private and one public government hospital) attached to a medical college in Mangalore, South India. The study participants included older persons who were defined as people aged ≥ 60 years seeking health care at the study hospitals and who provided informed consent to participate in the study. Patients who were seriously ill, nonambulatory, had known neuropsychiatric illness, were unable to verbally communicate with the investigators, and were unwilling to participate were excluded from the study. Patients who met the inclusion criteria and agreed to participate on a voluntary basis were subjected to a face-to-face interview using a validated and structured proforma.

The study was conducted for a duration of one month, during which data collection was carried out for a duration of 15 days. The sample size for the study was estimated on the basis of a previous study and calculated by the formula n = Z^2^*p*q/e^2^ [11], where ‘n’ represents the calculated sample size and Z represents the standard normal value for the 95% confidence interval, which is equal to 1.96. The prevalence of a high risk for malnutrition among older people in the previous study was 48.80% (p) [12]. The relative precision (e) was set at 20%. Considering a non response rate of 10%, the final sample size was calculated as 117. A process of convenience sampling was applied to select the study participants. The study used a validated, structured proforma for the purpose of data collection, which consisted of Sections such as, basic participant details such as age, sex, education, occupation, marital status, living status, and financial status and Mini-Nutritional Assessment Short-Form (MNA-SF) for nutritional assessment of older individuals which is already a validated questionnaire [13]. Socioeconomic status was assessed using the modified Kuppuswamy scale. Information regarding comorbid conditions was gathered. Face validity and the content validity has been carried out by the subject experts.

Mini-Nutritional Assessment Short-Form (MNA-SF) for nutritional assessment of older individuals [13]. The MNA-SF tool consists of six items and is simple to administer [14]. Of the six items, two items carry a maximum of score 3 and other four items carry a maximum score of 2. The minimum score for each item was score 0. Thus, the maximum score of the MNA-SF is 14. Interpretation of scores was performed as follows: score 0–7: malnourished; score 8–11: at risk of malnutrition; and score 12–14: normal nutritional status [14]. Anthropometric examination was performed for height and weight on the basis of the standard technique. Weight and height were measured using the standardized digital weighing scale and measuring tape respectively. Weight measured was approximated to the nearest 0.1 kg, and height measured was approximated to the nearest 0.1 cm. Weight and height were used to calculate the Body Mass Index of the study participants. The patients were interviewed and the collected data was entered into the Google Form for the ease of data collection. The downloaded data from google form was then transferred and analysed using IBM SPSS Statistics for Windows, version 29.0. Armonk, NY: IBM Corp [15]. Descriptive statistics such as the mean (standard deviation) and proportions were used to express the baseline characteristics and morbidity details of the overall study participants, whereas a chi-square test was performed to assess the various factors associated with the risk for malnutrition among the study participants. A p value of less than 0.05 was considered to indicate statistical significance.

The results were further analysed using univariate analysis to assess the factors associated with the risk of malnutrition. All the variables which showed a statistically significant result (p value less than 0.20) in the univariate analysis were included in the multivariable regression model to identify the factors which are independently associated with the risk of malnutrition or malnourished.

## Results

A total of 117 older people seeking health care in the two tertiary care hospitals attached to Kasturba Medical College, Mangalore participated. Among the 117 participants, 69 (59%) received health care from government hospitals, while 48 (41%) sought health care from private hospitals. The study included participants of both genders who participated almost equally. The mean age of the study participants was 65.7 *±* 6.1 years. The sociodemographic details of the study participants are described in the table below (Table 1).

**Table 1:** Basic details of the study participants (*n* = 117)

Variables	Frequency	Percentage
Gender		
Female	53	45.3
Male	64	54.7
Education status		
Graduate	4	3.4
High school certificate	20	17.1
Illiterate	33	28.2
Intermediate or Diploma	4	3.4
Middle school certificate	27	23.1
Primary school certificate	29	24.8
Occupation status		
Clerks	2	1.7
Craft and related trade workers	4	3.4
Elementary occupation	24	20.5
Legislators, senior officials and managers	1	0.9
Plant and machine operators and assemblers	2	1.7
Professionals	3	2.6
Skilled agricultural and fishery workers	25	21.4
Skilled workers and shop and market sales workers	21	17.9
Technicians and associate professionals	3	2.6
Unemployed	32	27.4
Marital status		
Married	83	70.9
Single	14	12.0
Widowed	20	17.1
Living status		
Alone	13	11.1
Only relatives	9	7.7
With children	21	17.9
With children and spouse	58	49.6
With spouse	16	13.7
Financial status		
Dependent	65	55.6
Independent	52	44.4

Table 2 describes the morbid conditions of the study participants. One-third of them (36.8%) were suffering from exclusively hypertension for which treatment was initiated. One-third of the total participants had only diabetes mellitus (35%). Approximately one-tenth of the participants had only dyslipidemia, thyroid conditions and heart diseases. Around 69.2% of the study participants had multiple morbidities. In terms of the screening using the MNA tool, 40.2% of the older population had a normal nutritional status, whereas the rest of them were either at risk or already having malnutrition (Table 3).

**Table 2:** Morbidity details of the study participants (*n* = 117)

Variables	Frequency	Percentage
Hypertension	43	36.8
Diabetes	41	35.0
Dyslipidemia	9	7.7
Thyroid disease	11	9.4
Heart disease	13	11.1
Multiple morbidity	81	69.2

*The participants may have had more than one morbidity condition

**Table 3:** Malnutrition assessment among the study participants (*n* = 117)

Variables	Frequency	Percentage
Normal nutritional status	47	40.2
At risk of malnutrition	51	43.6
Malnourished	19	16.2

As described in Tables 4, 58.1% of the participants were malnourished or at risk of malnourishment in the age group of less than 70 years and 64.5% in the age group 70 and above. A total of 58.4% of the participants with an education of up to middle school were malnourished or at risk of malnourishment, and 64.3% had an education of middle school or higher. Compared with 58.9% of the working population, 62.5% of the participants who are not working are malnourished. Among the participants in the lower/upper lower socioeconomic status category, 62.9% were malnourished or at risk of malnutrition, while 56.4% were in the lower middle/upper middle socioeconomic status category. 59.1% of married participants were malnourished or at risk of malnourishment, whereas 61.8% of unmarried, widowed or divorced participants were malnourished or at risk of malnourishment. A total of 76.9% of participants living alone were malnourished or at risk of malnourishment, while 57.7% of those living with spouses or family were malnourished or at risk of malnourishment. 50% of those who are financially independent and 67.7% of those who are financially dependent are malnourished or at risk of malnourishment.

**Table 4:** Association between factors and nutritional status among the study participants (*n* = 117)

Variables	MNA Score			Chi Square value	*P* value
	Malnourished	Normal	At risk		
Gender					
Female	9 (16.9%)	22 (41.5%)	22 (41.5%)	0.1722	0.917
Male	10 (15.6%)	25 (39.1%)	29 (45.3%)		
Education					
High school and above	5 (17.9%)	10 (35.7%)	13 (46.4%)	1.1827	0.88
Primary and Middle school	8 (14.3%)	22 (39.3%)	26 (46.4%)		
Illiterate	6 (18.2%)	15 (45.5%)	12 (36.4%)		
Occupation					
Working	10 (11.8%)	35 (41.2%)	40 (47.1%)	4.768	0.092
Not working	9 (28.1%)	12 (37.5%)	11 (34.4%)		
Marital status					
Married	14 (16.9%)	34 (41.0%)	35 (42.2%)	0.2464	0.88
Unmarried or widowed	5 (14.7%)	13 (38.2%)	16 (47.1%)		
Living status					
Alone or with relatives	3 (13.6%)	5 (22.7%)	14 (63.7%)	4.6633	0.09
With children, or spouse or both	16 (16.8%)	42 (44.2%)	37 (38.9%)		
Dependency status					
Dependent	14 (21.5%)	21 (32.3%)	30 (46.2%)	5.001	0.08
Independent	5 (9.6%)	26 (50.0%)	21 (40.4%)		
Hypertension					
No	11 (14.9%)	33 (44.6%)	30 (40.5%)	1.645	0.43
Yes	8 (18.6%)	14 (32.6%)	21 (48.8%)		
Diabetes					
No	11 (14.5%)	27 (35.5%)	38 (50.0%)	3.625	0.16
Yes	8 (19.5%)	20 (48.8%)	13 (31.7%)		
Dyslipidemia					
No	19 (17.6%)	42 (38.9%)	47 (43.5%)	2.159	0.34
Yes	0 (0.0%)	5 (55.6%)	4 (44.4%)		
Thyroid disease					
No	17 (16.0%)	45 (42.5%)	44 (41.5%)	2.609	0.27
Yes	2 (18.2%)	2 (18.2%)	7 (63.6%)		
Heart Disease					
No	17 (16.3%)	42 (40.4%)	45 (43.3%)	0.039	0.98
Yes	2 (15.4%)	5 (38.5%)	6 (46.2%)		
Socio economic status					
Upper or Middle	9 (16.4%)	24 (43.6%)	22 (40.0%)	0.6181	0.73
Upper Lower and Lower	10 (16.1%)	23 (37.1%)	29 (46.8%)		
Age group					
Less than 70 years	16 (18.6%)	36 (41.9%)	34 (39.5%)	1.7337	0.42
Above 70 years	3 (9.7%)	11 (35.5%)	17 (54.8%)		

Table 5 represent the univariate analysis and the multivariable analysis of the various factors associate with the risk of malnutrition. All the factors have been considered for the univariate analysis which showed a statistically significant result (p value less than 0.2) in few variables such as living status, dependency status, presence of diabetes and thyroid disease. There was a lower odds of risk of malnutrition among those older people who are independent and living alone. Similarly those who had diabetes mellitus and thyroid conditions had higher Odds of being at risk of malnutrition. Upon performing multivariable regression of the factors which had p value less than 0.2, living status and dependency status were found to be significant factors associated with risk of malnutrition.

**Table 5:** Univariate and Multivariable regression analysis of the factors associated with the risk of malnutrition (*n* = 117)

	OR (95% CI)	*P* value	aOR (95% CI)	*P* value
Gender				
Female	1.107 (0.527–2.325)	0.788	-	-
Male	Ref			
Education				
High school and above	0.667 (0.237–1.873)	0.442	-	-
Primary and Middle school	0.776 (0.325–1.854)	0.569	-	-
Illiterate	Ref			
Occupation				
Working	Ref			
Not working	0.857 (0.372–1.977)	0.718	-	-
Marital status				
Married	1.121 (0.494–2.541)	0.785	-	-
Unmarried or widowed	Ref			
Living status				
Alone or with relatives	0.371 (0.127–1.089)	0.071	0.31(0.1-0.959)	0.042
With children, or spouse or both				
Dependency status				
Dependent	Ref			
Independent	0.477 (0.225–1.013)	0.054	0.425(0.191–0.947)	0.036
Hypertension				
No	Ref			
Yes	0.600 (0.273–1.316)	0.202	-	-
Diabetes				
No	Ref			
Yes	1.728 (0.799–3.740)	0.165	1.78(0.781–4.054)	0.17
Dyslipidemia				
No	Ref			
Yes	1.964 (0.499–7.734)	0.334	-	-
Thyroid disease				
No	Ref			
Yes	0.301 (0.062–1.462)	0.137	0.255(0.05–1.294)	0.099
Heart Disease				
No	Ref			
Yes	0.923 (0.282–3.015)	0.894	-	-
Socio economic status				
Upper or Middle	Ref			
Upper Lower and Lower	0.762 (0.363–1.599)	0.472	-	-
Age group				
Less than 70 years	Ref			
Above 70 years	0.764 (0.326–1.790)	0.535	-	-

*Ref* Reference category, *OR* Odds Ratio, *aOR* adjusted Odds Ratio

Variables with p value less than 0.20 in univariate analysis were included in ther multivariable logistic regression model

## Discussion

The present study, which was conducted in the coastal Karnataka region of southern India, attempted to elucidate the nutritional profile of older individuals in this region of the country. This study highlights the substantial burden of malnutrition among older individuals visiting the hospital. Notably, approximately 40.2% of the study participants had good nutritional status, and 43.6% were at risk of developing malnutrition. Only one-sixth of the study participants (16.2%) were actually having malnutrition status as per assessments carried out using a standard tool. The univariate analysis performed showed that living status, dependency status, presence of diabetes and thyroid disease are the factors associated with risk of malnutrition which was later confirmed by multivariable analysis.

Few studies have explored similar outcomes across the globe. One of the studies conducted in the state of Rajasthan assessed nutritional status using the Mini Nutritional Assessment (MNA) scale. 11% of the older people were malnourished, 46% were at risk of malnutrition, and 42.4% had a normal nutritional status [16]. Malnutrition and the risk of malnutrition were more prevalent among the rural older people than among the urban older people. A higher prevalence of malnutrition was observed among individuals with poor socioeconomic status and those who were dependent on others. Malnutrition was more common among participants with a lower body mass index and was absent among those with a BMI > 23 kg/m². Nutritional status decreased with increasing age, with urban older people showing better nutritional status than rural older people did, as per a study conducted in Pondicherry [17]. 

Another study conducted in South India revealed that among the 190 older people included in the study, 19.47% were malnourished (MNA < 17) and 24.73% were at risk of malnutrition (MNA 17–23.5). The prevalence of malnutrition was high and significantly associated with sociodemographic factors, as well as the somatic and functional status of older population [18]. 

A similar study conducted by Andre et al. using a similar instrument revealed that 57.8% of older individuals were at risk of malnutrition and 28.4% were malnourished on the MNA scale. All malnourished participants had at least one chronic illness, and 87.6% were functionally dependent, indicating a high burden of malnutrition among the older people [19]. 

Although the results of the present study were not statistically significant on performing tests related to association, clinically significant changes were observed, as approximately two-thirds of the study population remained vulnerable to nutritional deficiency. Notably, traditional demographic factors such as sex, occupation or education did not significantly influence nutritional status but may indicate complex relationships among physiological senescence, psychosocial factors and functional capacity. Although chronic conditions were not associated with nutritional status, this does not necessarily diminish their effect on malnutrition. They act indirectly by reducing their appetite and altering their sleep habits, thereby making them ineligible to take up good amounts of nutrients. The multifactorial causation of the malnutrition cascade should be treated very carefully, and estimation of individual parameters may not yield many results.

### Recommendations

Age-specific interventions are essential for addressing malnutrition among older people, with routine geriatric screening facilitating early detection and timely dietary supplementation. Since malnutrition is observed across various educational and occupational groups, culturally appropriate nutritional education should begin earlier in the life course to promote healthy aging. Strengthening social security measures, including community feeding programs and livelihood-linked nutritional support schemes, can help reduce food insecurity among vulnerable older people. Addressing financial vulnerability through comprehensive community-based nutritional strategies is crucial for addressing the multiple determinants of malnutrition and ensuring dignity and quality of life in later years.

### Limitations

One of the main limitations of this study is that the findings cannot be generalized to a wider population, as the samples were selected using a convenience sampling technique from a single institution which has two hospitals (one public and one private) and represented a limited geographical area. The relatively small and localized sample from the hospital may not adequately reflect the true nutritional status of the broader older community and possibility of bias is present. Therefore, larger community-based studies conducted across diverse settings are necessary to generate more generalizable evidence and guide effective implementation strategies.

## Supplementary Information

Supplementary Material 1.

## Data Availability

The datasets used and/or analysed during the current study are available from the corresponding author on reasonable request.
