# Supplementary material for: Assessment of nutritional status among older people seeking health care in tertiary care hospitals in Mangalore
Source: BMC Geriatr. 2026 Jul 3;26:1054. doi: 10.1186/s12877-026-07950-5 (PMC13471594; doi:10.1186/s12877-026-07950-5)
Supplement: Supplementary file 1 [file 12877_2026_7950_MOESM1_ESM.docx]

**Interview questionnaire**

**Assessment of nutritional status among elderly persons seeking health care in tertiary care hospitals of Mangalore**

1. Serial Number:
2. Name of the hospital: a. Public Hospital	b. Private Hospital
3. Age (in years)…….. 4. Gender: Male/Female
5. Education of the participant: ……………………….
6. Occupation of the participant: …………………………………..
7. Education of the head of the family: ……………………….
6. Occupation of the head of the family: …………………………………..
7. Socio Economic status (As per Kuppuswamy Classification):

a. Class 1	b. Class 2	c. Class 3	d. Class 4	e. Class 5

8. Marital status: a. Single	b. Married	c. Widowed	d. Divorced
9. Living status: a. With spouse b. With children c. With spouse and children d. Only relatives e. Alone
10. Financial status: a. Dependent	b. Independent
11. Hypertension	a. Yes	b. No
12. Diabetes	a. Yes	b. No
13. Dyslipidemia	a. Yes	b. No
14. Thyroid disease	a. Yes	b. No	15. Heart disease	a. Yes	b. No
16. Any other disease (specify)………………….
17. Weight in kgs……………………… 18. Height in mts…………………………

19.Body Mass Index(BMI)…………………………..kg/m^2^

12-14 points: Normal nutritional status

8-11 points: At risk of malnutrition

0-7 points: Malnourished

MNA Score: __________

Interpretation of MNA score: __________
